# Lessons from a Rare Familial Dementia: Amyloid and Beyond

**DOI:** 10.13188/2376-922X.1000009

**Published:** 2015-08-03

**Authors:** Adam Cantlon, Carlo Sala Frigerio, Dominic M. Walsh

**Affiliations:** 1 Laboratory for Neurodegenerative Research, School of Biomolecular and Biomedical Science, Conway Institute, University College Dublin, Republic of Ireland; 2 Laboratory for Neurodegenerative Research, Ann Romney Center for Neurologic Diseases, Brigham and Women's Hospital and Harvard Medical School, Boston, USA

**Keywords:** Amyloid Bri, Amyloid β-protein, Amyloid β-protein precursor protein, Amyloid Dan, Alzheimer's disease, Familial british dementia, Familial danish dementia

## Abstract

Here we review the similarities between a rare inherited disorder, familial British dementia (FBD), and the most common of all late-life neurological conditions, Alzheimer's diseases (AD). We describe the symptoms, pathology and genetics of FBD, the biology of the BRI2 protein and mouse models of FBD and familial Danish dementia. In particular, we focus on the evolving recognition of the importance of protein oligomers and aberrant processing of the amyloid β-protein precursor (APP) - themes that are common to both FBD and AD. The initial discovery that FBD is phenotypically similar to AD, but associated with the deposition of an amyloid peptide (ABri) distinct from the amyloid β-protein (Aβ) led many to assume that amyloid production alone is sufficient to initiate disease and that ABri is the molecular equivalent of Aβ. Parallel with work on Aβ, studies of ABri producing animal models and in vitro ABri toxicity experiments caused a revision of the amyloid hypothesis and a focus on soluble oligomers of Aβ and ABri. Contemporaneous other studies suggested that loss of the ABri precursor protein (BRI2) may underlie the cognitive deficits in FBD. In this regard it is important to note that BRI2 has been shown to interact with and regulate the processing of APP, and that mutant BRI2 leads to altered cleavage of APP. A synthesis of these results suggests that a “two-hit mechanism” better explains FBD than earlier toxic gain of function and toxic loss of function models. The lessons learned from the study of FBD imply that the molecular pathology of AD is also likely to involve both aberrant aggregation (in AD, Aβ) and altered APP processing. With regard to FBD, we propose that the C-terminal 11 amino acid of FBD-BRI2 interfere with both the normal function of BRI2 and promotes the production of cystine cross-linked toxic ABri oligomers. In this scenario, loss of BRI2 function leads to altered APP processing in as yet underappreciated ways. Given the similarities between FBD and AD it seems likely that study of the structure of ABri oligomers and FBD-induced changes in APP metabolites will further our understanding of AD.

## Introduction

Dementia is a problem of immense proportions which afflicts over 36 million people [1]. Monogenic familial dementias account for a only a tiny percentage of the global dementia burden but study of these rare inherited disorders offer important insights about much more common conditions such as sporadic Alzheimer's disease (AD). Familial British dementia (FBD) is extremely rare, but shares many similarities with sporadic AD [2]. FBD is associated with a mutation on the *BRI2* gene [3] and the product of this gene (BRI2) is implicated in regulating the amyloid β-protein precursor (APP) [4,5]. The FBD mutation is also associated with the production of an aggregation-prone 34 residue long peptide, ABri. Below we review the symptoms, pathology and genetics of FBD, the biology of the BRI2 protein and its interaction with APP, mouse models of FBD and familial Danish dementia. We compare what is known about FBD and AD, and suggest some lessons that may be learned about AD based on concepts uncovered from the study of FBD.

## Familial British Dementia and Familial Danish Dementia

FBD was first described in a single family by Worster-Drought in 1933 [6-8] and subsequently in two other studies [9,10]. Descendants from all three families can be traced back to a couple born in England around 1780, with the second eldest daughter a common ancestor to both the Worster-Drought and Griffiths pedigrees and the youngest son the direct ancestor of the Love and Duchen pedigree [2]. Currently there are 372 individuals in the extended pedigree with approximately 50 individuals at risk of developing the disease [2,11].

FBD is typified by spastic tetra-paresis, dysarthria, loss of memory and dementia [8]. Affected individuals develop symptoms in the fifth decade of life and death occurs approximately ten years later [12]. The key histological features include parenchymal amyloid deposition, cerebral amyloid angiopathy (CAA), neurofibrillary degeneration and ischemic white matter damage [6,7]. Large diffuse plaques (up to 180 μm in diameter) which stain weakly with Congo red are numerous in the cerebellum, the cerebellar cortex, the dentate gyrus and the hippocampus. Smaller more strongly Congophilic positive plaques (up to 30 μm in diameter) are also found in the hippocampus [2,9], and appear highly similar to those found in AD. Amyloid associated proteins including amyloid P, apolipoprotein E and apolipoprotein J co-localize with these plaques [13], while GFAP-positive staining is evident surrounding larger plaques [12]. Inclusions of the trans-activation-responsive DNA-binding protein 43 (TDP-43) which occur in up to a quarter of all AD cases [14] have also been identified in FBD [15]. Systemic amyloid deposits are also present in the blood vessels of multiple peripheral tissues, including the myocardium and pancreas [16].

An even more rare disease related to FBD, familial Danish dementia (FDD), was described by Strömgren and colleagues in 1970, in a single family from Jutland, Denmark [17]. As of 2002, there were 13 affected individuals, across 5 generations [18]. FDD shares similarities to FBD and AD but also has certain unique symptoms. For instance, unlike AD or FBD, FDD patients often develop cataracts in their 30's and experience hearing loss in their 40's. Ataxia and dementia develop in the fifth and sixth decades of life and death typically occurs within 10 years [17]. Pathological features include severe CAA, neurofibrillary degeneration and ischemic white matter damage but in contrast to FBD and AD the parenchymal deposits in FDD are not readily stained with Congo red and have been described as “pre-amyloid” [18,19].

The clinical and pathological presentations of FBD can be so similar to AD that certain cases have been mistakenly described as atypical variants of AD [20]. Indeed a familial form of AD, associated with deletion of exon 9 of *Presenilin 1* (*PSEN1*) has a clinical presentation highly similar to FBD, with subjects developing spastic paraparesis prior to onset of dementia [21]. In AD, FBD and FDD intracellular neurofibrillary tangles (NFT) contain hyper-phosphorylated forms of the microtubule-associated protein, tau. The NFTs in AD, FBD and FDD are composed of paired helical filaments (PHF) and have identical immunohistochemical properties. PHFs isolated from FBD, FDD and AD brain also contain the same ratios of 3- and 4-repeat tau and are phosphorylated at common epitopes [18,22,23]. Together, these similarities suggest that the changes seen in tau may involve a common mechanism.

## Genetics of FBD and FDD

**Figure 1:**
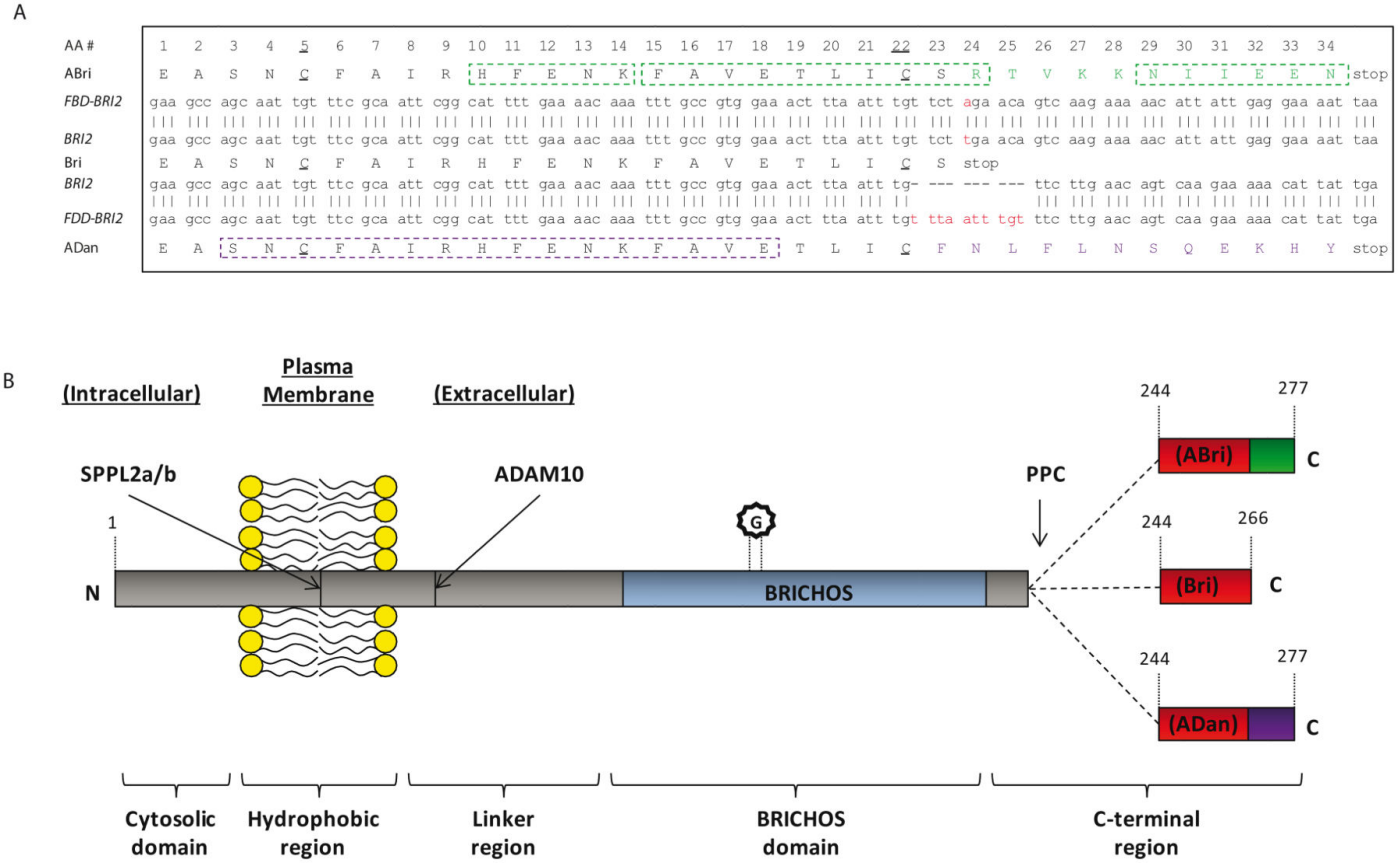
Nucleotide sequence and schematic representation of BRI2 proteins (A) Nucleotide sequences of the last 34 (ABri), 23 (Bri) and 34 (ADan) amino acids of the FBD-BRI2, BRI2 and FDD-BRI2 genes, respectively. Amino acids are numbered (AA #) with E244 as 1 and cystines underlined. A point mutation, (t for a at codon 267) is highlighted in red on the FBD-BRI2 nucleotide sequence and converts the in frame, stop codon into an arginine extending the open reading frame (ORF) by 11 amino acids. A 10 nucleotide duplication (TTTAATTTGT between codons 265 and 266) is highlighted in red on the FDD-BRI2 nucleotide sequence which converts serine 266 to phenylalanine and extends the ORF by 11 amino acids. The individual protein sequences derived from tryptic digestion of either FBD or FDD plaques are indicated with green and purple dashed boxes, respectively. (B) Schematic representation of BRI2 proteins. The N-terminal (N), the C-terminal (C), an N-glycosylation site at position 170 (G) and the BRICHOS proteins are indicated. The general nomenclature applied to BRICHOS domains are denoted with horizontal brackets below the schematic. Sites of proteolysis by proprotein-like convertase (PPC), the disintegrin and metalloprotease ADAM10 and signal peptide peptidase-like proteases (SPPL2a and SPPL2b) are shown with arrows.

When partially purified extracts of leptomeningeal CAA deposits and parenchymal FBD plaques were examined by denaturing SDS-PAGE the principal component was found to migrate with an apparent molecular weight of ∼4 kDa. A combination of tryptic digestion and protein sequencing identified 3 peptide fragments (HFENK, FAVETLICSR and NIIEEN) (Figure 1A). BLAST analysis using a database of expressed sequence tags yielded a perfect match with HFENK and a near perfect match with FAVETLICSR. The only discrepancy between FAVETLICSR and the previously reported expressed sequence tag was that the FAVETLICSR contained an arginine residue where a stop codon had been reported (Figure 1A). The expressed sequence tag corresponded to a 266 residue long protein known as BRI2 or Integral membrane protein 2B (Itm2B). Furthermore, DNA sequence encoding the tryptic fragment NIIEEN displayed perfect homology with the 3′-untranslated region immediately prior to the next in frame stop codon of the *BRI2* sequence (Figure 1A). Subsequent DNA sequencing revealed that 7 individuals with FBD had a point mutation (*t* for *a*) at codon 267 in the *BRI2* gene. The FBD mutation converts the normal stop codon in *BRI2* into the codon for an arginine residue and extends the open reading frame to include an additional 11 amino acids producing the 277 amino acid long FBD-BRI2 protein. The FBD mutation introduces an XbaI cleavage site and allows easy identification of individuals carrying the FBD-BRI2 mutation. Importantly, unafected individuals from the same kindred lacked the *t* for *a* nucleotide substitution, demonstrating that the mutation is pathogenic and not merely a benign polymorphism. Polyclonal antibodies raised to the amyloid British (ABri) peptide purified from FBD plaques (antibody-547), or to a synthetic peptide (CTVKKNIIEEN) spanning residues 24-34 of the ABri molecule (antibody-338) recognize FBD plaques. This evidence directly links the mutation which produces the FBD-BRI2 protein to the novel peptide that constitutes the major protein component of FBD plaques [3].

Soon after identification of the FBD mutation, the genetic defect associated with FDD was discovered. The XbaI restriction site was absent from the sequence isolated from the FDD kindred indicating that the *tga* to *aga* mutation was not present in FDD. Instead DNA sequencing identified a 10 nucleotide duplication (TTTAATTTGT between codons 265 and 266), immediately prior to the normal in frame stop codon (Figure 1A). This replaces the terminal residue of BRI2 (serine) with a phenylalanine and extends the ORF generating a 277 amino acids long protein which we refer to as FDD-BRI2. Both FDD-BRI2 and FBD-BRI2 are 11 residues longer than BRI2 but the additional residues share no sequence homology. The final 12 residues of FDD-BRI2 differ from BRI2, while the final 11 residues of FBD-BRI2 and BRI2 differ (Figure 1A). As with FBD, antibodies raised to the synthetic amyloid Danish peptide (ADan) stain amyloid plaques in FDD brain [19]. The ability to identify mutant sequences by reverse genetics, use the sequence to produce synthetic peptides and generate antibodies specific for the mutant sequence, which subsequently stain plaques provides definitive proof of the link between the mutations which produces FBD-BRI2 and FDD-BRI2 and the novel peptides found in FBD and FDD plaques [3,19].

## Te BRI2 Protein

BRI2 is a type II transmembrane protein (Figure 1B), the gene for which is located on human chromosome 13q14.2 and is ubiquitously expressed [24]. In mice, BRI2 protein is highest in the cerebellum and midbrain regions, followed by the lung and heart [25]. The BRI2 protein is capable of forming homodimers, both in cell culture and mouse brain. This occurs through formation of a disulphide bond between two adjacent cysteines, located at amino acid position 89 in the BRI2 sequence and via non-covalent interactions [26]. In addition N-glycosylation of BRI2 at position 170 (Figure 1B) is believed to be important in its trafficking to the cell surface [24,27].

The BRI2 protein contains an evolutionary conserved domain, known as the BRICHOS domain. This is approximately 100 residues long and is found in 309 proteins. The term BRICHOS is derived from 3 of these proteins, BRI2, Chondromodulin-I and surfactant protein C (SP-C). BRICHOS domain-containing proteins share common structural features including a cytosolic region, a hydrophobic domain, a linker region, a BRICHOS domain and a C-terminal region (Figure 1B) [28,29]. BRICHOS domain-containing proteins can be divided into twelve groups based on sequence homology. In eleven of these groups, which include the BRI2 protein, the C-terminal region has a high propensity to form β-sheet structure. It has been hypothesized that the BRICHOS domain acts as an intra-molecular chaperone and interacts with C-terminal β-sheet rich regions. Thus, the BRICHOS domain may stabilize proteins and prevent formation of β-sheet-rich aggregates [28]. SP-C is the only BRICHOS-containing protein which lacks a C-terminal region; however unlike proteins in the other 11 BRICHOS groups, the hydrophobic domain of SP-C has a high propensity to form β-sheet structure. SP-C is expressed in the lungs where it augments the adsorption and spreading of surfactant lipids and is crucial for normal lung function [30]. Mutations in the SP-C gene (*SFTPC*) are associated with interstitial lung disease. Two separately occurring mutations, L188Q and ΔExon4 occur within the SP-C BRICHOS domain and lead to accumulation of intracellular protein aggregates [31,32]. Expression of SP-C^L188Q^ or SP-C^ΔExon4^ in cell culture, results in the formation of Congo red positive, amyloid-like deposits suggesting that these mutations disrupt the native chaperone function of the BRICHOS domain [33]. In a complementary study, *in vitro* aggregation experiments indicate that the wild type BRICHOS domain can ameliorate formation of β-sheet-rich aggregates, inhibiting Aβ fibril formation at sub-stoichiometric concentrations [34-36]. Therefore the BRICHOS domain may have a dual role, both as an intra-molecular chaperone and an inter-molecular inhibitor of amyloid formation. It is interesting to note that processing of BRI2 releases a soluble fragment which includes the BRICHOS domain (Figure 1B) and could serve as an extracellular inhibitor of amyloid formation.

The exact physiological role of BRI2 in the central nervous system is still unknown; however proposed functions for BRI2 are similar to those attributed to APP. For instance, over-expression of BRI2 induces elongation of neurites in a neuroblastoma cell line suggesting it may be involved in neuronal differentiation [37]. Moreover, expression of BRI2 is increased during acute trauma and is concentrated at nerve terminals proximal to ischemic lesions and in dystrophic neurites adjacent to senile plaques implying that BRI2 may be involved in stress response pathways [38]. The finding that BRI2 can form homodimers and is present on the cell surface has led some to speculate that BRI2 may act as a receptor [26].

Processing of BRI2 occurs at 3 sites: (1) near the carboxyl-terminal by a proprotein-like convertase (PPC), (2) at the juxtamembrane by the disintegrin and metalloprotease ADAM10, and (3) within the plasma membrane by signal peptide peptidase-like proteases (SPPL2a and/or SPPL2b) (Figure 1B) [39-41]. Several PPC's including furin, paired basic amino acid cleaving enzyme 4 (PACE4), lymphoma proprotein convertase (LPC) and proprotein convertase 5/6 (PC 5/6) are capable of processing BRI2. Of these potential BRI2 cleaving enzymes, furin appears to be the most effective [39,40]. When CHO cells were transfected with cDNA encoding BRI2 or FBD-BRI2 cleavage of these proteins generated Bri and ABri peptides, respectively. However neither peptide was detected when furin deficient CHO cells were used, indicating that furin is required for liberation of Bri and ABri [39]. Interestingly, FBD-BRI2 appears to be a better substrate for PPC than BRI2, suggesting that under certain circumstances the C-terminal extension may result in enhanced PPC processing of FBD-BRI2. However, BRI2 has an atypical furin cleavage site _238_KGIQKREA_245_ and in a separate study attenuation of furin activity with a specific inhibitor did not alter BRI2 processing in N2a neuroblastoma cells [37]. Furthermore furin activity is restricted to the trans-Golgi network whereas cleavage of the BRI2 C-terminal has been observed earlier in the cis-medial-Golgi [37]. Therefore it appears that more than one PPC may contribute to the physiological cleavage of BRI2 and that cleavage of FBD-BRI2 is influenced by the differential cellular expression of PPCs

## ABri and ADan Aggregation and Toxicity

Accumulation of aggregation-prone peptides in the brain is believed to be the primary event driving pathogenesis in AD and etiologically similar entities [42-44]. Indeed FBD plaques are surrounded by reactive microglial and proximal to dystrophic neurites [12,23]. However, neither temporal nor spatial progression of amyloid plaques correlate well with clinical progression in AD [45,46] and burgeoning evidence indicates that soluble non-fibrillar assemblies of aggregation-prone peptides may constitute the most clinically relevant species [47].

ABri, ADan and Bri have two cysteine residues (C5 and C22) (Figure 1A) and therefore have the potential to form either intra-molecular or inter-molecular cystine bonds. Intra-molecular disulphide bonds would result in the formation of cyclized monomer, whereas inter-molecular disulphide bonds could give rise to oligomers of varying length. Oxidized cyclized ABri monomer has been detected in extracts from FBD brain [3] and a ladder of bands corresponding to ABri monomers, dimers, trimers and higher molecular weight oligomers has been detected on SDS-PAGE from brain derived material [16]. Initial work with synthetic ABri and ADan focused on whether these peptides could aggregate and if so, to determine whether fibrils and/or prefibrillar intermediates were capable of compromising cell viability. Since ABri and ADan are capable of forming distinct structures, due to disulphide bond formation, whether oxidation affects structure and cell viability was also investigated. When assessing peptide aggregation, it is essential to begin with isolated monomer, since preformed assemblies can enhance aggregation. Likewise, when comparing the effects of oxidation, fully defined species should be used since untreated peptides likely contain a mixture of both oxidized and reduced species. It is important to note that the experiments described below generally utilized synthetic ABri and ADan in an undefined state i.e. the degree of oxidation and/or aggregation was not rigorously determined prior to initiation of the studies. Thus, it is difficult to make definitive interpretations regarding certain data.

Electron microscopic studies found that synthetic ABri formed irregular, short, tortuous fibrils with average diameters of 5 nm and of unspecified length and periodicity [39]. Under quiescent conditions ABri fibril formation occurred at acid pH (pH 4.9), while amorphous-like aggregates were detected at neutral (pH 7.3) and alkaline pH (pH 8.4). However during peptide synthesis and/or subsequent HPLC purification, use of trifluoroacetic acid is common, which may lead to acidification of the peptide [48]. Thus, ABri dissolved in neutral (pH 7.3) or alkaline (pH 8.4) buffers must transverse its isoelectric point (pI) ∼6.85 [49]. This can result in peptide precipitation and may explain why amorphous ABri aggregates were detected at pH's above the pI of ABri. Nonetheless, AFM studies of ABri at pH 4.9 revealed that several distinct morphological structures emerged during aggregation. ABri initially formed small spherical aggregates (0.5-1.5 nm in height), which self-associated into short protofibrils (1.5-2.3 nm height and 250 nm in length), then longer protofibrils (500 nm in length), and finally mature fibrils (200-800 nm in length and 4.0-6.0 nm in height) [50]. Under acid conditions (pH 4.8), oxidized ADan (pI 6.05) also formed protofibrillar species prior to the emergence of amyloid fibrils [51,52]. As seen with ABri, oxidized ADan did not form fibrils when dissolved in neutral (pH 7.0) or alkaline (pH 9.0) buffers. Again this was likely due to isoelectric precipitation. Deliberately reduced ADan primarily formed non-fibrillar, spherical or dome-shaped (30-40 nm in height) aggregates under acid, neutral and alkaline conditions [51,52].

Oxidized ABri dissolved in 100 mM Tris-HCl pH 9 and aged for 3 weeks at 37 °C contained a mixture of species including oligomers (which migrated on SDS-PAGE as a series of increasing molecular weight bands), protofibrils and mature fibrils, whereas freshly dissolved oxidized ABri primarily contained oligomers. Both freshly prepared and aged solutions of oxidized ABri but not reduced ABri were found to induce apoptotic cell death as measured by Annexin V/propidium iodide staining, in a neuroblastoma cell line (SHSY-5Y). The potency of these species was dependent on assembly size with non-fibrillar, oligomeric ABri species more toxic than preparations of ABri which contained protofibrils and mature fibrils [53]. Although the authors speculated that oligomers were formed from cyclized monomers, it was unclear whether the oxidized ABri formed inter- or intra-molecular disulphide bonds and an equally likely alternative explanation would be that the oligomers were formed by inter-molecular cross-links. Indeed the inability of strong denaturants (DMSO or HFIP) to disaggregate these oligomers is more consistent with the formation of covalently cross-linked oligomers [54]. In other studies both oxidized and reduced ADan species were found to induce apoptotic cell death in SHSY-5Y cells with oligomeric ADan having greater bioactivity than non-fibrilar ADan aggregates [51,55].

Application of synthetic ABri and ADan in cell culture models clearly demonstrates that mixtures containing pre-fibrillar, oligomeric species compromise cell viability to a greater extent than mature fibrils. However it is important to note that these toxicity experiments had two major drawbacks: (1) immortalized undifferentiated cell lines and not neurons were used, and (2) supra-physiological concentrations of synthetic peptide, in some instances as high as 300 μM were applied [52]. Recently we used ABri preparations with defined oxidation and aggregation state and assessed their effect on synaptic plasticity using organotypic hippocampal slices, and on the viability of dissociated hippocampal neurons [56]. This allowed us to discover that the oxidation state of ABri determined both its aggregation pathway and toxic activity. Cyclization of ABri and Bri produced monomers which showed no propensity to assemble. Conversely, reduced ABri and reduced Bri aggregated forming thioflavin T-positive amyloid fibrils. However, neither cyclized monomers nor reduced aggregates exerted significant toxic activity. ABri formed inter-molecular disulphide bonds to a greater degree than Bri and the formation of covalently stabilized ABri oligomers was associated with toxicity. These results suggest that the 11 amino acid extension to the C-terminal of Bri causes a shift in the type of disulphide bonds formed and that covalently cross-linked ABri oligomers interact with neurons to compromise their function and viability.

## Pyroglutamation of ABri and ADan Peptides

The N-terminal residue of Bri, ABri and ADan is glutamate and pyroglutamation (pE) of these peptides increases hydrophobicity and decreases their solubility in aqueous solutions. *In vitro* studies using a continuous thioflavin-T assay indicate that pyroglutamation of ABri and ADan markedly increases aggregation propensity [57,58]. Thus pyroglutamation of ABri could lead to the accelerated aggregation and deposition and may explain why pE-ABri is so prevalent in FBD plaques [16]. However, since plaques can persist for decades, it is also possible pyroglutamation occurs after deposition. In this scenario pyroglutamate would protect against proteolysis and in part might explain why deposited ABri is not proteolysed. With regard to whether pyroglutamate formation occurs before or after deposition the discovery that extracellular soluble ABri has an unmodified glutamate residue at the N-terminus strongly suggests that pyroglutamate formation occurs post deposition [16].

## Mouse Models of FBD and FDD

Cell culture models are used extensively in neurodegenerative research as they offer a closed system in which to test mechanistic hypotheses, pharmacological interventions and are amenable to high-throughput analysis. Indeed, neuronal loss is a common feature of AD and FBD and well defined peptide preparations may be used in these models to test the relationship between structure and toxicity, over a short period of time. However, the earliest manifestation of disease in AD and FBD is a progressive decline in cognitive function which cannot be recapitulated in cell culture models. Historically mice have been utilized as preclinical model systems in which to test cognitive status and to determine how pathology correlates with cognition [59]. Several models of FBD and FDD have been developed including transgenic *FBD-BRI2*, *FDD-BRI2* and *BRI2* mice, knock-in *FBD-BRI2* and *FDD-BRI2* mice and knock-out *BRI2* mice (Table 1), as well as numerous crosses. However, it is important to stress that none of the BRI2 mouse models fully recapitulate all the features of FBD or FDD.

The first generation FBD mouse models (MoPrPmtBRI2 and Thy-1.2-mtBRI2) transgenically over expressed *FBD-BRI2* (Table 1) but early FBD-BRI2 lines did not deposit ABri, nor did they exhibit any overt behavioral phenotype [60]. Immunoprecipitation of Thy-1.2-mtBRI2 brain extracts and plasma indicated that cleavage of FBD-BRI2 and secretion of ABri had occurred in this model but at low levels. Subsequent Tg-FDD and ADanPP7 mice (Table 1), developed FDD-like histological changes, including widespread, amyloid deposition, CAA, neuritic dystrophy and microglial activation [61,62]. Tg-FDD mice develop pathology at 7 months of age, with CAA localized to the cerebellar vessels (leptomeningeal), progressing to the neocortex, hippocampus, thalamus and olfactory bulb. Vascular ADan deposits co-localized with amyloid P-component and ApoE. Thioflavin S (ThS) positive plaques were detected in the cerebral cortex and diffuse ADan parenchymal deposits were observed throughout the brain with the latter also staining with A11, an antibody reported to bind to soluble oligomers of aggregation-prone peptides [63]. Tg-FDD mice develop phospho-tau-immunopositive deposits throughout the neuropil of the cerebral cortex and hippocampus. Tg-FDD mice, crossed with tau transgenic mice (TauP301S) expressing mutant human tau (P301S) develop enhanced NFT pathology, tau phosphorylation and tau truncation when compared to TauP301S littermates. These effects occur prior to widespread amyloid deposition indicating that soluble, non-fibril forms of ADan or expression of *FDD-BRI2* augments tau pathology in these mice [64]. Importantly no enhanced ADan pathology was detected in Tg-FDD/TauP301S mice when compared to Tg-FDD littermates. Together this data suggests that the neurofibrillary pathology evident in FDD occurs downstream of altered mutant-BRI2 processing, probably as a consequence of the formation of toxic oligomers. Congruent with profound cerebellar ataxia seen in patients with FDD, Tg-FDD mice develop abnormal gait and posture after 12 months [18,62]. However, this phenotype makes testing memory and learning difficult and the relationship between amyloid burden and cognition has not been assessed in Tg-FDD mice.

ADanPP7 rapidly accumulate ADan in the parenchyma and developed leptomeningeal CAA by 2 months of age [61]. As in the human condition, parenchymal ADan deposits in ADanPP7 mice are ADan-immunoreactive but generally do not stain with Congo red. Dystrophic neurites are detected in the vicinity of ADan deposits but there was no gross neuron loss. At ∼20 months ADanPP7 develop an anxiety-related phenotype, accompanied with a marked decrease in body weight. Also starting at ∼20 months ADanPP7 mice developed immune mediated hair loss (alopecia) and excessive curvature of the spine (kyphosis) phenotypes believed to result due to non-CNS effects of *FDD-BRI2*. The Morris water maze test was used to assess whether these animals developed deficits in spatial learning and memory and at ∼20 months ADanPP7 mice took longer to locate the hidden platform, compared with age-matched non-transgenic controls. However, ADanPP7 mice did not swim as fast as age-matched controls. Therefore the deficit observed in the cued navigation task may have resulted from a motor rather than cognitive impairment. Furthermore, ADanPP7 did not manifest cognitive deficits at ages when there was extensive amyloid deposition; a finding that clearly uncouples amyloid deposits and impaired cognition.

Although transgenic *mutant-BRI2* animals deposit amyloid, which is a histological hallmark of FBD and FDD, they are unlikely to replicate disease features which result from loss of *BRI2* function. On the other hand BRI2 knock-in mice suggest that loss of BRI2 function does contribute to cognitive dysfunction. FBD_KI_ and FDD_KI_ mice (Table 1) express a single copy of the *FBD-BRI2* or *FDD-BRI2* gene respectively and these animals produce reduced amounts of mature BRI2 and exhibit early and profound cognitive deficits [65-67]. The finding that mature BRI2 levels are reduced in mutant-BRI2 knock-in mice is congruent with FBD and FDD which are heterozygous conditions in which preliminary evidence suggest the levels of mature BRI2 are decreased by ∼50% [65,68]. FBD_KI_ and FDD_KI_ mice develop age-dependent hippocampal memory deficits in the novel object recognition (NOR) paradigm and the radial-arm water maze (RAWM) and exhibit impairments in long-term potentiation (LTP), a correlate for learning and memory (Table 1). Transgenic expression of *BRI2* in FDD_KI_ mice alleviates memory and LTP impairments, whereas *BRI2* haplodeficient mice (*BRI2*^+/-^) develop deficits in object recognition memory, spatial working memory and LTP [65,66]. Together these findings strongly suggest that loss of mature BRI2 is likely to play an important role in FBD and FDD [69]. *Bri2* null mice are viable and fertile but have not been studies in detail [70].

Proteolytic processing of APP is enhanced in FDD_KI_ mice and APP metabolites, including Aβ, were significantly increased in extracts derived from an FDD patient [68]. Intriguingly, BRI2 has been proposed to regulate processing of APP. Specifically, there is evidence that BRI2 binds to APP and modulates its processing [4,5]. Expression of BRI2 and APP deletion mutant constructs in HEK cells reveal that residues 46-106 of BRI2 bind to the juxtamembrane and membrane spanning domains of APP (residues 648-719, based on APP_751_ numbering) [4]. Down regulation of endogenous BRI2 in HEK293APP cells increases secretion of APPsα, APPsβ, Aβ_40_, and Aβ_42_ suggesting that BRI2 may inhibit APP processing by sterically restricting access by the secretases, which mediate APP proteolysis. Additionally, transgenic mice expressing human wild-type BRI2 (MoPrP-BRI2) crossed with transgenic APP mice (CRND8) produce significantly less APPsα, APPsβ, Aβ_40_, and Aβ_42_ than littermate CRND8 controls. Thus expression of BRI2 *in vitro* and *in vivo* appears to regulate processing of APP [70]. Evolving data suggest that APP processing is more complex than previously thought [71,72] but for the purposes of this review we will restrict consideration to only the canonical amyloidogenic and non-amyloidogenic pathways [73]. In the amyloidogenic pathway β-secretase cleaves APP to release a large ectodomain fragment known as APPsβ, while simultaneously producing a membrane-bound, 99 amino acid long, C-terminal fragment, βCTF. The βCTF is subsequently processed by γ-secretase to yield the APP intracellular domain (AICD) and Aβ peptides with varying C-termini. In the non-amyloidogenic pathway cleavage of APP within the Aβ domain by α-secretase generates APPsα and the 83 amino acid long, αCTF. The αCTF is also a substrate for γ-secretase yielding AICD and p3 peptides with varying C-termini. Use of pharmacological inhibitors of γ-secretase (GSI) and β-secretase (BSI) indicate that a metabolite of APP other than Aβ may be responsible for the cognitive deficits in FDD_KI_ mice. Specifically, administration of the GSI, compound-E, worsened memory deficits in 6 month old FDD_KI_ mice [74], whereas BSIs alleviated synaptic and behavioral deficits in FDD_KI_ mice [75]. Together these data suggest that derivatives of APP generated by β-secretase cleavage are responsible for cognitive deficits in FDD_KI_ mice [75].

Further evidence linking loss of BRI2 function to APP comes from studies in which FBD_KI_ and FDD_KI_ mice were crossed with mice expressing only one APP allele (APP^+/-^). The resulting FBD_KI_/APP^+/-^and FDD_KI_/APP^+/-^ animals exhibited no LTP or memory deficits [69,76]. The requirement of two *APP* alleles to be expressed in order for these deficits to be observed is consistent with APP metabolites mediating concentration-dependent effects on synaptic plasticity and memory. However, the identity of the APP derivative or collection of derivatives that are altered by BRI2 dysfunction and contribute to impaired cognition is as yet unclear. From studies beyond FBD and FDD, there is evidence that certain APP derivatives are directly toxic while others are neurotrophic. For example, in mouse models the accumulation of β-cleaved APP (APPsβ) and/or β-carboxyl terminal fragments (β-CTF) affect LTP [77] and memory acquisition [75]. Moreover, amyloidogenic APP-CTFs are neurotoxic *in vitro* and can cause AD-like neuropathology *in vivo*. In contrast, certain secreted forms of APP and CTFs may have physiologically important roles [78] such that an imbalance in the relative levels of these could lead to pathologic signaling [79,80]. Therefore mutations in the *BRI2* gene may corrupt normal BRI2 function and mediate their effects through altered production of one or more APP derivatives.

## Conclusion

### A two hit model best explains FBD/FDD and AD

**Figure 2:**
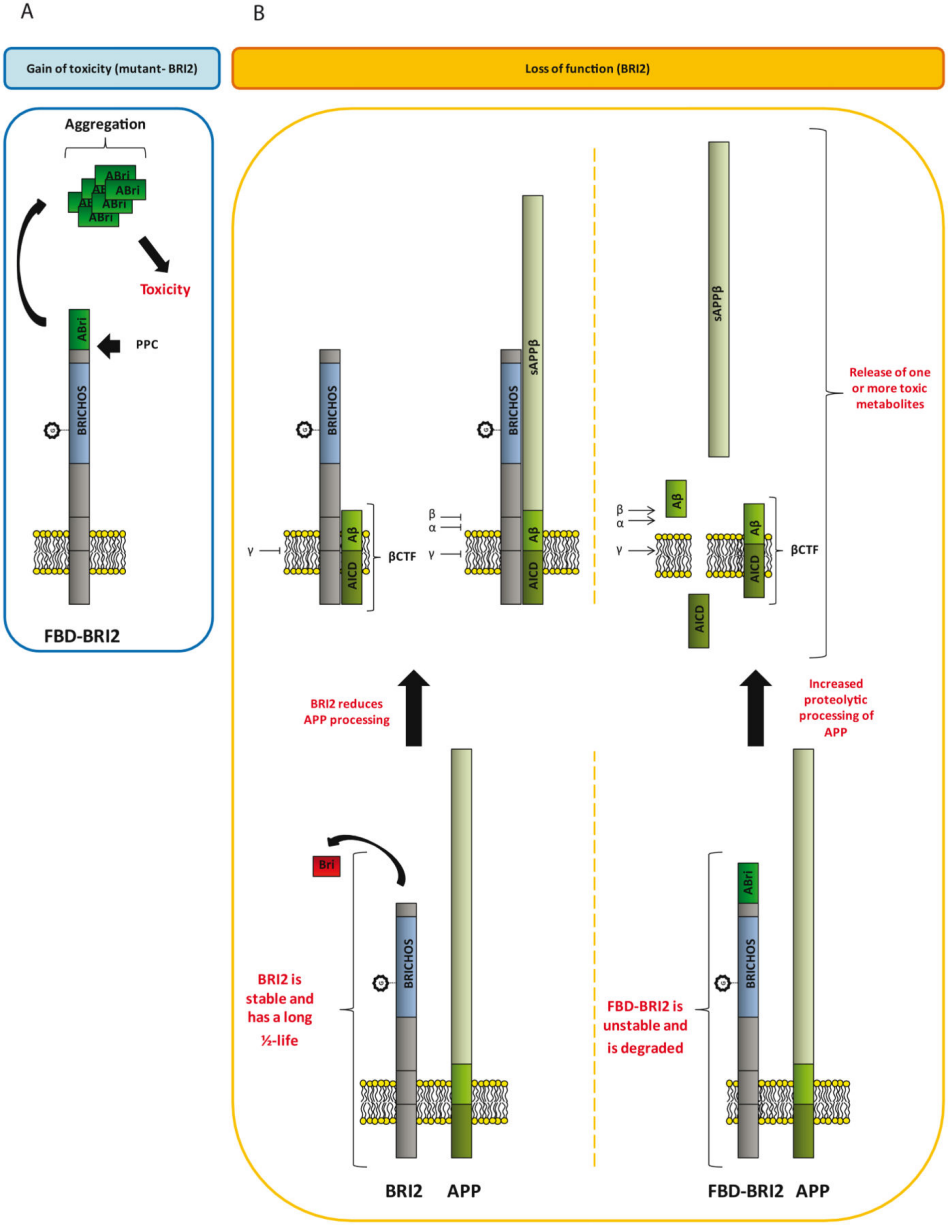
Pathogenic mechanisms that may operate in FBD (A) Gain of toxic activity hypothesis. Accumulation of ABri leads to aggregation and formation of toxic species. Schematic representation of FBD-BRI2 showing production of ABri, its ensuing aggregation and subsequent toxicity. Note, the ABri assembly shown is for illustrative purposes only. It is important to consider that assemblies of different sizes and structures may have toxic activity. Accumulation of ABri leads to aggregation and formation of toxic species. (B) Loss of function hypothesis. Schematic representation of BRI2, FBD-BRI2 and APP. BRI2 is processed by PPC to form mature BRI2. Mature BRI2 forms complexes with APP, inhibiting APP processing by α-, β- and γ-secretase (ῑ). Mature BRI2 is also capable of forming complexes with βCTF inhibiting γ-secretase (ῑ). The FBD mutation renders FBD-BRI2 unstable such that most FBD-BRI2 is degraded and therefore more APP is accessible to proteolytic processing by α-, β- and γ-secretase (→), and this leads to formation of one or more toxic metabolites e.g. βCTF, AICD and sAPPβ.

Clearly, the mechanisms underlining disease in FBD and FDD are complex and multifaceted. Nonetheless, the data extant are considerable and have given rise to two competing hypotheses to explain the causation of FBD and FDD (Figure 2). One hypothesis focuses on a toxic gain of function, in which mutations give rise to ABri or ADan that form toxic aggregates (Figure 2A). *In vitro*, aggregation studies have shown that ABri and ADan form oligomers, protofibrils and fibrils [51,53], while under similar conditions Bri does not readily aggregate and the assemblies Bri does form are not toxic [56]. Further, Tg-FDD and ADanPP7 mice exhibit age-dependent amyloid deposition associated with neuritic dystrophy, microglial activation, aberrant tau phosphorylation and deficits in motor control [61,62]. Both of these observations are consistent with a toxic gain of function (Figure 2A).

However, Tg-FDD and ADanPP7 mice appear cognitively normal and show no signs of gross neuronal loss despite appreciable amyloid burden. Albeit, neuronal loss in Tg-FDD and ADanPP7 mice was measured relative to non-transgenic, age-matched controls. Since BRI2 has been implicated in neurite elongation and neuronal differentiation [37], it will be important to assess whether enhanced neurogenesis potentially masks neuronal loss in Tg-FDD and ADanPP7 mice. Indeed, hematoxylin and eosin staining of Tg-FDD cortical sections reveal neurons with a pyknotic appearance (irreversible condensation of chromatin), indicative of cells undergoing necrosis or apoptosis [62]. This phenotype is not evident in age-matched non-transgenic controls and suggests elevated levels of cell death in Tg-FDD mice.

The second hypothesis that tries to explain the molecular basis of mutant-BRI2 diseases proposes that FBD and FDD are a consequence of loss of BRI2 function (Figure 2B). Mutations in the *BRI2* gene appear to destabilize the BRI2 protein, which is targeted for degradation, leading to deregulation of APP processing and formation of one or more toxic APP metabolites (Figure 2B). This theory is strongly supported by FBD_KI_, FDD_KI_ and BRI2^+/-^ mouse models, which develop profound synaptic and cognitive impairments, recapitulating the early memory loss which is characteristic of BRI2-linked dementias [65,66]. Preliminary data suggest that the levels of mature BRI2 are reduced in the brains of FBD and FDD patients and in brains of FBD_KI_ and FDD_KI_ mice. Transgenic expression of BRI2 and haplo insufficiency of APP rescues impairments in FBD_KI_ and FDD_KI_ cognition. These findings are consistent with a loss of BRI2 function since both approaches equalize the ratio of mature BRI2 and APP. With regard to transgenic models of mutant BRI2 if memory impairment is mediated by loss of mature BRI2, then this would not be apparent in such mice because these models express normal levels of endogenous murine BRI2 and high levels of mutant-BRI2.

**Figure 3:**
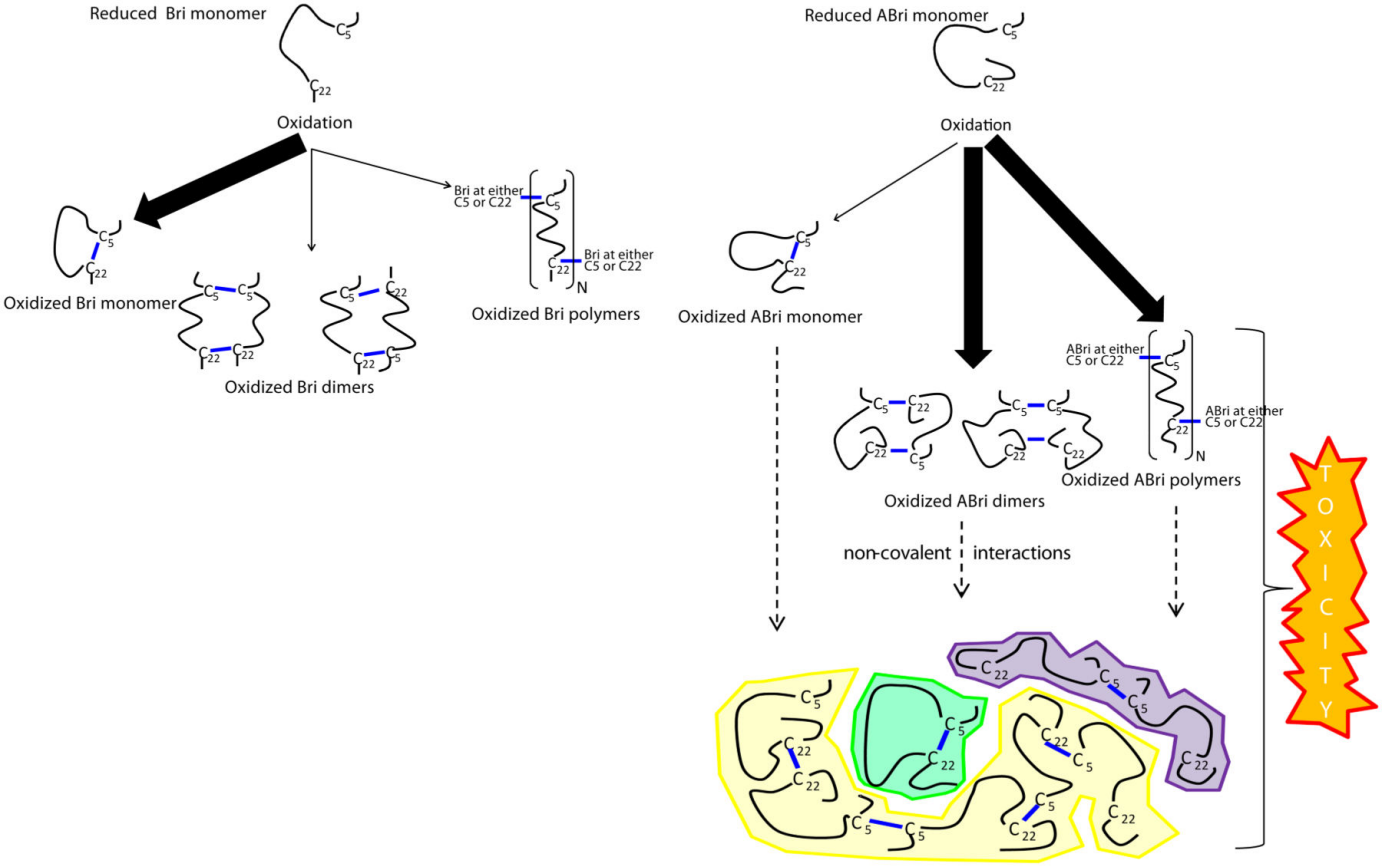
C-terminal extension of Bri peptide facilitates the generation of covalently cross-linked neurotoxic ABri species (A) The 23 amino acid long wild type Bri peptide spontaneously oxidizes forming intra-molecular disulphide bonds (thick arrow); producing a compact cyclized structure that exhibits no propensity for aggregation and neither alters nerve cells viability nor LTP. Although theoretically possible, the data extant indicate that Bri does not form significant amounts of inter-molecular cross-linked multimers (→). (B) In contrast, ABri oxidizes forming both inter- (→) and intra-(thin arrow) molecular disulphide bonds. As with Bri, cyclized ABri appears not to aggregate, whereas cross-linked ABri oligomers are found in ThT-positive amyloid structures. (C) Importantly, mixtures of oxidized ABri that include ABri monomer (shaded in green), non-sedimentable cross-linked oligomers (shaded in purple and yellow) and amyloid fibrils (not shown), inhibit LTP and are potent neurotoxins. These results suggest that decreasing the formation of inter-molecular disulphide bonds in ABri would prevent or reduce ABri toxicity. Figure adated from Cantlon et al. 2015.

The loss of BRI2 function hypothesis does not accommodate all of what we know about FBD and FDD. Specifically, if loss of BRI2 is the root cause of these diseases then the pathology and symptoms in FBD and FDD should be identical. This is not the case. As we reviewed above, FBD and FDD have distinct clinical and pathological features. Moreover, given that ABri and ADan deposition is a prominent feature in FBD and FDD, respectively, maturation of mutant-BRI2 (i.e. liberation of ABri and ADan) must occur, at levels sufficient to induce amyloidosis and as a consequence, significant amounts of mature functional BRI2 must be produced. Of course the toxic gain of function and toxic loss of function hypothesis are not mutually exclusive. Indeed we propose that etiology of FBD is best explained by both a toxic gain and loss of function. At a molecular level FBD is directly attributable to the increased ability of the cysteines within the ABri domain of FBD-BRI2 to form intermolecular cross-links that act: (i) to destabilize FBD-BRI2 and make it less prone to proteolytic maturation, and (ii) to give rise to the formation of covalently stabilized toxic ABri oligomers (Figure 3) [56]. In this model reduced levels of mature BRI2 contribute to impaired synaptic plasticity, and cystine-linked ABri oligomers cause aberrant changes in tau and consequent neuronal loss. Simultaneously, unoxidized ABri aggregates and forms amyloid plaques. A major advantage of this model over the toxic loss of function model is that the differential toxicity of ABri and ADan can explain the phenotypic differences in these diseases. The two hit model involving: (1) aberrant protein aggregation, and (2) altered APP processing seems to best explain FBD and by analogy indicates that both Aβ aggregation and altered APP processing are likely to contribute to AD pathogenesis.

Clearly, further studies will be required to validate this overarching hypothesis. In particular it will be important to determine whether oxidized ABri can induce disease-relevant changes in tau, and whether the preliminary reports of loss of FBD-BRI2 maturation in end-stage FBD brain are also detected in FBD iPSC-derived neurons. Notwithstanding the need for further research, the study of FBD has already generated important information which may offer new opportunities for a better understanding of the most common of all human dementias, sporadic AD. Not least the elegant studies by D'Adamio and colleagues [65,75,76] highlight the need to consider the many different derivatives that are produced from APP. On the other hand our observation that only oxidized oligomers of ABri are toxic to neurons suggests that high resolution analysis of ABri oligomers may provide important insights on non-covalent Aβ oligomers.

## Figures and Tables

**Table 1:** BRI2 and mutant-BRI2 mouse models.

Gene	Model Name	Promotor	Background	Expression relative to wt BRI2	Histological changes	Age of onset of histological changes	Method of detecting histological changes	Behavioral paradigms	Behavioral deficit	Age of onset of behavioral deficit	Reference
***BRI2***	MoPrP-BRI2	MoPrP	B6/D2/SW	∼1.5 times expression	No histological changes evident	N/A	N/A	N/A	N/A	N/A	Pickford [60]
***FBD-BRI2***	MoPrP-mtBRI2	MoPrP	B6/D2/SW	∼2.5 times expression	No histological changes evident	N/A	N/A	N/A	N/A	N/A	Pickford [60]
***FBD-BRI2***	Thy-1.2-mtBRI2	Thy-1.2	B6/D2/SW	∼2.5 times expression	No histological changes evident	N/A	N/A	N/A	N/A	N/A	Pickford [60]
***FDD-BRI2***	Tg-FDD	MoPrP	C57BL/6J	Unspecified	CAA, amyloid deposition and microglial activation	7 months	Anti-ADan AB's, ThS dye, Anti-GFAP AB's	N/A	Unfeasible due to abnormal gait and posture	N/A	Vidal [62]
***FDD-BRI2***	ADanPP7	MoPrP	C57BL/6J	Several fold higher	CAA, amyloid deposition, microglial activation and neuritic dystrophy	2 months	Anti-ADan AB's, ThS & Congo red dye, Anti-GFAP AB's	Open field, Morris water maze (MWM)	Increased anxiety	Open field 18-20 months	Coomaraswamy 2010
***FDD-BRI2***	FDDKI	Endogenous BRI2 promoter	C57BL/6J	Normal expression	No histological changes evident	N/A	N/A	Open field, Long-term potentiation (LTP), Novel object recognition (NOR), radial-arm water maze (RAWM)	No deficit in open field, reduced LTP, deficit in NOR, deficit in RAWM	LTP 11-13 months, NOR 5-8 months, RAWM 5-11 months	Giliberto [65] Tamayev [65]
***FBD-BRI2***	FBDKI	Endogenous BRI2 promoter	C57BL/6J	Normal expression	No histological changes evident	N/A	N/A	Open field, NOR, RAWM, contextual fear-conditioning (CoFC), cued fear-conditioning (CuFC)	No deficit in open field, deficit in NOR, deficit in RAWM, deficit in CoFC, no deficit in CuFC	NOR 10 months, RAWM 9 months, CoFC 9 months	Tamayev [65]
***BRI2***	BRI2+/-	Endogenous BRI2 promoter	C57BL/6J	0.5 times expression	No histological changes evident	N/A	N/A	Open field, LTP, NOR, RAWM, CoFC, CuFC	No deficit in open field, deficit in NOR, deficit in RAWM, deficit in CoFC, no deficit in CuFC	NOR 7 months, RAWM 7 months, CoFC 7 months	Matsuda [66] Tamayev [65]

Not applicable (N/A)

## Abbreviations

ABri: Amyloid Bri
ADan: Amyloid Dan
AD: Alzheimer's Disease
APP: Amyloid β-protein Precursor Protein
Aβ: Amyloid β-protein
CAA: Cerebral Amyloid Angiopathy
FBD: Familial British Dementia
FDD: Familial Danish Dementia
GFAP: Glial Fibrillary Acidic Protein
LTP: Long Term Potentiation
NFT: Neurofibrillary Tangles
PHF: Paired Helical Filaments
SDS-PAGE: Sodium Dodecyl Sulphate Polyacrylamide Gel Electrophoresis
SEC: Size Exclusion Chromatography
TDP-43: Trans-activation-responsive DNA-binding Protein 43
TT: Tioflavin-T
β-CTF: β-C-Terminal Fragment
